# Development of a prediction model for mortality in infants undergoing therapeutic hypothermia for neonatal encephalopathy

**DOI:** 10.1038/s41372-025-02547-z

**Published:** 2026-01-05

**Authors:** Jill M. Mitchell, Clare L. Rodrigues, Margo Dunworth, Julie Mc Ginley, Laura O’Byrne, Roisin Hall, Paul Corcoran, Indra San Lazaro Campillo, Peter Mc Kenna, John R. Higgins, Joye McKernan, Ali S. Khashan, Gillian M. Maher, John Murphy, Brian H. Walsh, Richard A. Greene, Fergus P. McCarthy

**Affiliations:** 1 Department of Obstetrics and Gynaecology, University College Cork, Cork, Ireland; 2 National Women and Infants Health Programme, Cork, Ireland; 3 National Perinatal Epidemiology Centre, Cork, Ireland; 4 INFANT Research Centre, Cork, Ireland; 5 School of Public Health, University College Cork, Cork, Ireland

**Keywords:** Paediatrics, Epidemiology, Risk factors, Pregnancy outcome

## Abstract

**Objective:**

To develop and internally validate a model predicting neonatal mortality in infants with neonatal encephalopathy requiring therapeutic hypothermia (TH), using national data.

**Study design:**

Data from 385 infants treated with TH across 19 hospitals (2016–2021) were analysed. Multivariable logistic regression with backward stepwise selection was applied. Discrimination was assessed using the C-statistic, with internal validation by bootstrapping. The THERM (Therapeutic Hypothermia Early Risk Model for Mortality) tool was developed to calculate individualised mortality risk.

**Results:**

Forty-six infants (11.9%) died within 28 days. Four predictors were retained: prelabour Caesarean section, adrenaline use, base excess ≤–22 mmol/L, and seizures during the first day of life. The model demonstrated excellent discrimination [optimism-adjusted C-statistic 0.885 (95% CI: 0.827–0.936)].

**Conclusions:**

Four routinely collected variables predicted mortality in infants undergoing TH. The THERM tool provides a practical resource for clinicians, enabling personalised risk assessment and supporting parental counselling during the first day of life.

## Introduction

Neonatal encephalopathy (NE) is a significant cause of morbidity and mortality in term infants [1, 2]. Therapeutic hypothermia (TH) is the standard treatment for moderate to severe NE and has been shown to reduce mortality and improve neurodevelopmental outcomes in survivors [3, 4]. Despite its effectiveness, there remains a need for accurate early prediction models to enhance parental counselling.

Among outcomes following NE, death is the most immediate to prognosticate and carries profound implications for both clinicians and families [5]. Accurate early prognostication of death allows parents to begin processing the possibility of goals of care discussions or loss, and to make preparations per family, cultural, or religious needs [5]. Prediction models can aid this process by offering individualised, evidence-based mortality risk estimates. Importantly, such tools are intended to support parental counselling and emotional preparation, not to guide decisions about initiating or continuing therapeutic hypothermia, which should be made based on established clinical criteria.

Ambalavanan et al. developed a prediction model for neonatal death in infants with NE, using American data from over 20 years ago [6]. Advances in clinical practices may limit the accuracy of this model in a modern setting, and it has not been validated. More recent studies have been region-specific or rely on magnetic resonance imaging (MRI), which limits their applicability in high-income settings or for early clinical use [7–9]. Glass et al. developed a prediction model for the composite outcome of death or severe neurodevelopmental impairment, but did not provide performance metrics for death as an isolated outcome and included death up to 2 years of life [10].

This study aims to develop a prediction model using early neonatal risk factors to predict neonatal mortality in infants with neonatal encephalopathy requiring TH, using data from 19 maternity hospitals. The model is designed to facilitate clearer, more informed parental counselling in the early days of life, without influencing clinical decision-making regarding the use of TH.

## Methods

### Study population

We used data from paired mother-infant medical records of infants who received TH across 19 maternity hospitals in the Republic of Ireland between 2016 to 2021, collected through the National Neonatal Therapeutic Hypothermia Development Project.

Data access approval was granted from the National Neonatal Steering Committee, the National Perinatal Epidemiology Centre, the National Women and Infant Health Programme, and the National Clinical Programme for Paediatrics and Neonatology for the use of anonymised data for this project.

Infants diagnosed with neonatal encephalopathy and requiring TH were included in the study. Those with genetic anomalies were excluded. This study followed the TRIPOD guidelines (available at https://www.tripod-statement.org).

### Variable selection

We selected routinely measured predictors by considering antenatal factors, delivery factors, early neonatal evaluations, and laboratory results. Our approach combined expert opinions from neonatologists and obstetricians, alongside a review of existing literature, taking into account the available data [2, 4, 6–8, 11–14]. The variables; parity, maternal age, body mass index (BMI), employment status, smoking status at booking, maternal medical condition in current pregnancy, previous Caesarean section, presence of meconium, gestational age at delivery, sex of infant, birth weight, mode of delivery, birth status, occurrence of acute perinatal event, time of birth, day of birth, 5-min Apgar score < 3, establishment of spontaneous respiration during resuscitation, use of adrenaline during resuscition, chest compressions, intubation, base excess ≤ −22 mmol/L, pH ≤ 6.70, seizures during day one of life were considered as potential predictors. Parity was recorded as the number of completed pregnancies ≥24 weeks and recategorised as nulliparous or multiparous. Maternal age, measured in years, was retained as a continuous variable and was also assessed as a categorical variable (≤24 years, 25–34 years, 35–39 years and ≥40 years). BMI was categorised into ≤24.9 kg/m^2^, 25.0–29.9 kg/m^2^ and ≥30 kg/m^2^. Employment status was categorised into employed, unemployed/student, and homemaker. Smoking status at booking was categorised as non-smoker and smoker. Maternal medical condition in pregnancy was defined as hypertensive disorders (including pre-eclampsia, pregnancy-induced hypertension, and essential hypertension), diabetes mellitus (either gestational or pre-existing), or thyroid disease (hypothyroidism or hyperthyroidism) in the current pregnancy. Mode of delivery was categorised as spontaneous vaginal delivery, operative vaginal delivery, Caesarean section after the onset of labour, or prelabour Caesarean section. Descriptive statistics were employed to analyse the category “prelabour Caesarean section” further to differentiate elective prelabour Caesarean sections and emergency prelabour Caesarean sections, whereby the Cesarean section was performed before the onset of labour, due to concerns for the well-being of either the mother or the baby. Birth status was defined as inborn or outborn. TH is exclusively provided in the four tertiary NICUs in Ireland. All other maternity sites transfer eligible newborns to one of these four units to receive TH [15]. “Inborn” was defined as birth at a site performing TH, while “outborn” included all infants born in an external location and transferred to the tertiary units for TH. The variables: ‘presence of meconium, 5-min Apgar score < 3, intake of a spontaneous breath during resuscitation, use of adrenaline during resuscitation, chest compressions, and intubation’ were also categorised separately as yes/no. Similarly, the variable “occurrence of an acute perinatal event” was also binary (yes/no), with perinatal event defined as umbilical cord prolapse, uterine rupture, antenatal haemorrhage, shoulder dystocia, or severe fetal heart rate abnormality [4]. Thresholds for base excess (≤−22 mmol/L) and pH (≤6.70) were derived from previously published research [6]. These measurements included umbilical artery and venous blood gases, as well as the first blood gas analysis from the infant within the first hour of life. The variables “seizures during day one of life, intake of a spontaneous breath during the initial resuscitation and use of adrenaline during resuscitation” were categorised separately as yes/no. Seizure activity during the first day of life was classified following review of medical notes by the study team. Infants were classified as having seizures if there was documented clinical seizure activity during the first day of life, confirmed by electroencephalogram (EEG) when available, and initiation of antiepileptic treatment. Diagnoses were made by senior neonatologists (consultants or senior registrars).

### Outcome measure

The outcome of interest was neonatal mortality, defined as neonatal death within the first 28 days of life, consistent with the World Health Organization definition of neonatal death [16].

### Statistical analysis

Statistical analysis was performed using Stata BE 18.5, SPSS version 29, and Microsoft Excel 16.90.2. Demographic factors were examined using descriptive analysis. Unadjusted logistic regression analysis examined associations between candidate predictors and the odds of neonatal death. Significant variables (p < 0.1) were entered into the multivariable logistic regression analysis. Variables with more than 15% missing data were excluded from the analysis; however, separate multivariable analyses were subsequently performed to assess the effect of these variables on the model’s predictive ability.

### Initial prediction model

A multivariable logistic regression with backward stepwise selection (*p* ≥ 0.1 for exclusion) was used to develop the initial prediction model [17]. All candidate predictors were included, and the least statistically significant predictors were removed one by one [17, 18]. We used Little’s test, utilising the *mcartest* command, to assess the assumption of missingness being completely at random (MCAR) [19]. A complete case analysis of all variables included was conducted to address missing values [20].

### Simplified prediction model

We then developed a simplified prediction model by removing one candidate variable at a time, completely excluding it from the candidate set, prior to running the backward stepwise selection. This iterative process continued until we obtained the simplest model that retained its predictive ability.

### Sample size

The sample size calculation was performed using the *pmsampsize* command. Assuming an outcome event proportion (prevalence) of 0.12, a target shrinkage factor of 0.85, a c-statistic of 0.81 [6–8], and 10 candidate predictors/categories, a minimum sample size of 353 (with 42 events) would be required to minimise overfitting [21].

### Model performance and internal validation

Model performance was evaluated by examining overall fit, discrimination, and calibration. Overall fit was assessed with the Brier Score and Cragg & Uhler’s (Nagelkerke) *R*². Discrimination was evaluated using the area under the ROC C-statistic. Calibration was assessed using calibration-in-the-large (CITL) and calibration slope (C-slope). Bootstrapping was performed for internal validation (with 100 repetitions) to assess overfitting and calculate the optimism-adjusted C-statistic, CITL, and C-slope. Supplementary information 1 (A1) provides additional details on the accepted thresholds for model performance metrics.

### Development of an interactive prediction tool

To facilitate the practical application of our prediction model, we developed an interactive Excel-based tool called the THERM tool (Therapeutic Hypothermia Early Risk Model for Mortality). The tool incorporates variables identified in the final multivariable logistic regression model, with each variable weighted according to its corresponding regression coefficient, reflecting its relative contribution to the overall risk. The tool computes the total log-odds using the logistic regression formula and transforms it into a probability using the inverse logit function. Additionally, an explanatory sheet provides clear definitions and coding for each variable, ensuring ease of use and accessibility for clinicians.

### Confusion matrix

To provide additional clinical context, we used the simplified prediction model, selected for its parsimony and clinical applicability, to classify infants as predicted to survive or die at a 50% probability threshold, and constructed a confusion matrix to compare predicted with observed outcomes.

## Results

### Descriptive statistics

Characteristics of the study participants are presented in Table 1. A complete descriptive analysis of the cohort, including maternal demographics and medical history, labour and delivery details, neonatal resuscitation, and biochemical characteristics, is documented in Tables A1 to A3.

**Table 1:** Characteristics of study participants.

Variable	All	Neonatal death		*p* value
	*N* (%)	No *N* (%)	Yes *N* (%)	
Parity	385	339	46	0.319
Primiparous	228 (58.7)	197 (58.1)	29 (63.0)	
Multiparous	159 (41.3)	142 (41.9)	17 (37.0)	
Not documented	0			
Sex of infant	385	339	46	**0.013**
Male	214 (55.6)	196 (57.8)	18 (39.1)	
Female	171 (44.4)	143 (42.2)	28 (60.9)	
Not documented	0			
Birth weight	385	339	46	0.249
≤2499 g	27 (7.0)	25 (7.4)	2 (4.3)	
2500–2999 g	69 (17.9)	59 (17.4)	10 (21.7)	
3000–3499 g	106 (27.5)	88 (26.0)	18 (39.1)	
3500–3999 g	118 (30.6)	107 (31.6)	11 (23.9)	
≥4000 g	65 (16.9)	60 (17.7)	5 (10.9)	
Not documented	0			
Mode of delivery	385	339	46	**0.005**
Spontaneous vaginal	92 (23.9)	84 (24.8)	8 (17.4)	
Operative vaginal	123 (31.9)	116 (34.2)	7 (15.2)	
Prelabour Caesarean section	82 (21.3)	65 (19.2)	17 (37.0)	
Caesarean section after onset of labour	88 (22.9)	74 (21.8)	14 (30.4)	
Not documented	0			

**Fig. 1:**
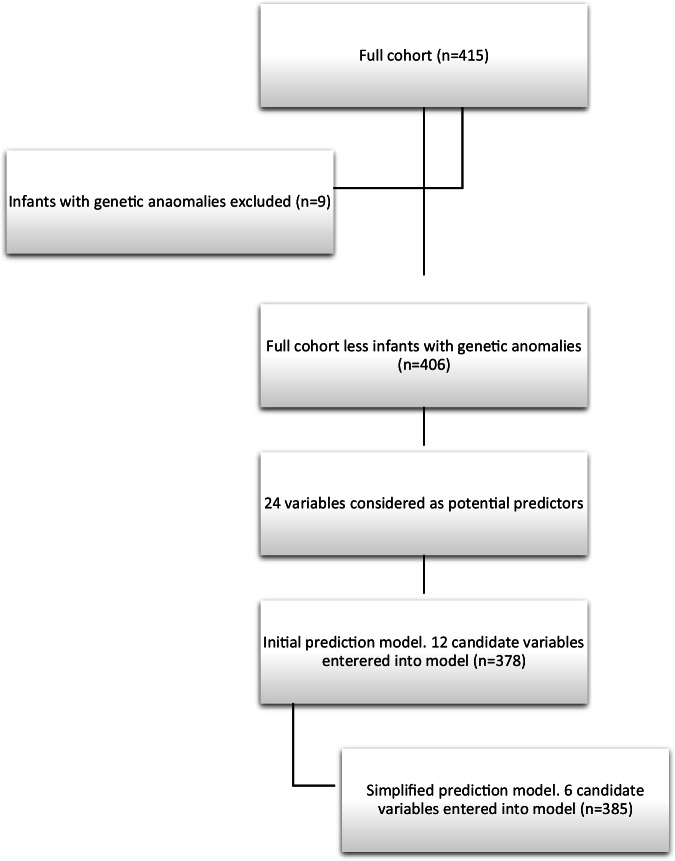
Graphical overview of methodology and sample size. Figure 1 summarises cohort exclusions, candidate variables considered, and the number of variables entered into the initial and simplified prediction models, with the sample size included in each model.

Out of 415 infants who required TH, nine were excluded due to genetic anomalies. In the initial prediction model, 6.9% of the data for candidate variables were missing, while 5.1% were missing in the simplified model. Little’s test indicated that the missing data did not deviate from the missing completely at random (MCAR) assumption (*p* = 0.908). The complete case analysis resulted in a final cohort of 378 infants for the initial prediction model, among whom 44 (11.6%) died, and 385 infants for the simplified prediction model, among whom 46 (11.9%) died (Fig. 1). All deaths occurred within the first 28 days of life; no additional deaths were recorded up to two years of follow-up. Descriptive statistics were performed for 385 mother–infant pairs included in the simplified model.

Of the included mothers, 58.7% (*n* = 228) were primiparous, and the majority (89.5%, *n* = 342) were White. Most deliveries occurred at term with 2.7% (*n* = 11) delivered before 36 weeks, 5.7% (*n* = 23) delivered between 36 + 0 and 36 + 6 weeks, 68.0% (*n* = 261) between 37 + 0 and 40 + 6 weeks, and 23.4% (*n* = 90) at or beyond 41 weeks (with 0.3%, *n* = 1 not documented).

Regarding mode of delivery, 23.9% (*n* = 92) had a spontaneous vaginal delivery, 31.9% (*n* = 123) had an operative vaginal delivery, 22.9% (*n* = 88) underwent a Caesarean section after labour onset, and 21.3% (*n* = 82) had a prelabour Caesarean section.

Most prelabour Caesarean sections in this cohort were emergency procedures (97.6%, *n* = 80). Acute perinatal events occurred in 75.1% (*n* = 289) of cases overall, rising to 90.2% (*n* = 74) among prelabour Caesareans. Rates were also high for Caesareans after labour onset (85.2%), operative vaginal deliveries (79.7%), and lower for spontaneous vaginal deliveries (45.7%). Differences across delivery modes were statistically significant (*p* < 0.001; Table A4).

### Unadjusted logistic regression analysis

The variables; sex of infant, presence of meconium, occurrence of an acute perinatal event, mode of delivery (specifically prelabor Caesarean section), use of adrenaline during resuscitation, chest compressions, 5 min Apgar score < 3, establishment of spontaneous respiration during resuscitation, intubation, base excess ≤ −22 mmol/L, pH ≤ 6.7 and seizures during day one of life were significant in the unadjusted logistic regression analysis (*p* < 0.1). The following variables were not statistically significant at *p* < 0.1: parity, body mass index, employment status, smoking status at booking, maternal condition in the current pregnancy, previous Cesarean section, gestational age at delivery, birth status, time of birth, day of birth, and maternal age.

### Initial prediction model

The initial analysis identified four variables as the best combined predictors of neonatal mortality: mode of delivery (prelabour Caesarean section), use of adrenaline during resuscitation, base excess ≤ −22 mmol/L, and seizures during the first day of life (Table 2).

**Table 2:** Best combined predictors of neonatal mortality in infants requiring therapeutic hypothermia, initial model.

Variable	Coefficient (95% CI)	N (%)	OR (95% CI)
Mode of delivery			
Spontaneous vaginal delivery	-	88 (23.3)	Ref
Prelabour Caesarean section	0.84 (-0.01, 1.68)	78 (20.63)	2.31 (0.99, 5.36)
Use of adrenaline			
No	-	320 (83.1)	Ref
Yes	2.18 (1.38, 2.97)	65 (16.9)	8.81 (3.97, 19.57)
Base excess ≤ −22 mmol/L			
No	-	316 (83.60)	Ref
Yes	1.62 (0.81, 2.44)	62 (16.4)	5.06 (2.25, 11.42)
Seizures on first day of life			
No	-	260 (68.78)	Ref
Yes	1.18 (0.37, 2.97)	118 (31.22)	3.24 (1.45, 7.27)
Constant	−4.03 (−4.83, −3.24)		0.02 (0.01, 0.04)

The model included spontaneous vaginal, operative vaginal, prelabour Caesarean section, and Caesarean section after labour onset; only prelabour Caesarean section was statistically significant and retained.

*Simplified Prediction Model*. For the simplified model, we removed one candidate variable at a time, completely excluding it from the candidate set, before running the multivariable logistic regression with backwards stepwise selection. This resulted in the inclusion of the candidate variables: mode of delivery, use of adrenaline during resuscitation, establishment of spontaneous respiration during resuscitation, intubation, base excess ≤ −22 mmol/L, and seizures during day one of life into the multivariable logistic regression model. The same four variables were identified in the simplified model as in the initial model (mode of delivery [prelabour Caesarean section], use of adrenaline during resuscitation, base excess ≤ −22 mmol/L, and seizure during day one of life) (Table 3).

**Table 3:** Best combined predictors of neonatal mortality in infants requiring therapeutic hypothermia, simplified model.

Variable	Coefficient (95% CI)	*N* (%)	OR (95% CI)
Mode of delivery			
Spontaneous vaginal delivery	-	92 (23.9)	Ref
Prelabour Caesarean section	0.86 (0.27, 1.69)	82 (21.3)	2.36 (1.03, 5.43)
Use of adrenaline			
No	-	320 (83.1)	Ref
Yes	2.20 (1.41, 3.00)	65 (16.9)	9.04 (4.09, 20.01)
Base excess ≤ −22 mmol/L			
No	-	322 (83.6)	Ref
Yes	1.67 (0.87, 2.48)	63 (16.4)	5.33 (2.38, 11.97)
Seizures on first day of life			
No	-	263 (68.3)	Ref
Yes	1.19 (0.38, 1.99)	122 (31.7)	3.28 (1.47, 7.33)
Constant	−4.01 (−4.89, −3.29)		0.02 (0.01, 0.04)

The model included spontaneous vaginal, operative vaginal, prelabour Caesarean section, and Caesarean section after labour onset; only prelabour Caesarean section was statistically significant and retained.

### Prediction tool

These four predictors were used to develop the THERM tool (Supplementary Material 2). For example, when a hypothetical case with a prelabour Caesarean section, adrenaline use during resuscitation, and base excess ≤ −22 mmol/L was entered, the tool generated a predicted mortality probability of 67%. The predicted probability of death increases to 87% when all four risk factors are present.

### Model performance and internal validation

For the initial model, the overall performance was good, with a Brier Score of 0.069 and Cragg & Uhler’s *R*² score of 0.43 [22–24]. The original apparent C-statistic was 0.900 (95% CI 0.854, 0.945), indicating excellent discriminative ability [25]. Bootstrapping adjusted the C-statistic to 0.873 (95% CI 0.816, 0.917), supporting the model’s capability to predict neonatal mortality effectively (Table 4).

**Table 4:** Assessment of model performance.

	Original apparent		Optimism	Optimism adjusted	
Initial model					
Discrimination					
C-statistic	0.900 (0.854, 0.945)		0.027	0.873 (0.816, 0.917)	
Calibration					
C-Slope	1.000 (0.760, 1.240)		0.139	0.861 (0.610, 1.110)	
CITL	0 (−0.391, 0.391)		−0.031	−0.031 (−0.465, 0.401)	
Overall fit					
Brier Score	0.069				
Cragg & Uhler’s *R*²	0.439				
Simplified Model					
Discrimination					
C-statistic	0.903 (0.860, 0.948)	0.018			0.885 (0.827, 0.936)
Calibration					
C-Slope	1.000 (0.765, 1.235)	0.099			0.901 (0.652, 1.194)
CITL	0 (−0.390, 0.390)	−0.002			−0.002 (−0.344, 0.480)
Overall fit					
Brier Score	0.069				
Cragg & Uhler’s *R*²	0.459				

*CITL* calibration-in-the-large, *C-slope* calibration slope.

Similarly, the simplified model performed well, with a Brier Score of 0.069 and Cragg & Uhler’s *R*² score of 0.459. The original apparent C-statistic was 0.903 (95% CI, 0.860–0.948), demonstrating the excellent discriminative ability of the model [25]. The original apparent C-statistic was 0.903 (95% CI 0.860–0.948), and after bootstrapping, the adjusted C-statistic remained high at 0.885 (95% CI 0.827–0.936) (Table 4). The miscalibration in CITL and C-slope was small in both the initial and simplified models, suggesting that overfitting was unlikely to be an issue.

In the unadjusted analysis, the variable “presence of meconium” was significant (OR = 1.84, 95% CI: 0.91–3.70, *p* = 0.09). However, due to 16.5% missing data, it was excluded from the primary multivariable analysis. A separate multivariable logistic regression model that included the variable’ presence of meconium’ did not improve the model’s predictive ability (AUC = 0.892, 95% CI: 0.840–0.944; see Table A5).

The calibration plots (Figs. A1 and A2) suggest that the average model predictions closely match the observed outcomes across ten groups of patients (i.e., deciles of risk were used as cut-off points to compare observed and expected probabilities within groups of individuals), indicating good calibration for both models. Most deciles were clustered at the lower end of the risk spectrum, reflecting the majority of individuals having low predicted probabilities of the event. The Lowess smoother highlighted minimal miscalibration at higher predicted probabilities; however, these estimates are based on limited data, as indicated by the sparse spike plot at higher risk levels.

At a 50% probability threshold, the simplified model correctly identified 329 of 354 infants predicted to survive (92.9%) and 21 of 31 infants predicted to die (67.7%) (Table A7).

## Discussion

### Principal findings

This study developed and internally validated a risk prediction model for neonatal mortality in infants with neonatal encephalopathy undergoing therapeutic hypothermia. We identified four variables routinely collected during the early neonatal resuscitation period to predict mortality in this population. These variables included mode of delivery (prelabour Caesarean section), use of adrenaline during resuscitation, base excess ≤ −22 mmol/L, and the presence of seizures during day one of life. The concordance of the same four predictors in both the initial and simplified models demonstrates their association with neonatal mortality in infants undergoing TH, reinforcing the credibility and stability of the THERM tool for individualised risk assessment. The association between prelabour Caesarean section and neonatal mortality is likely due to the high percentage of infants that had an acute perinatal event within this category. Notably, nearly all prelabour Caesarean sections were emergency procedures rather than elective procedures, meaning that the Caesarean section was performed before the onset of labour, due to concerns for the well-being of either the mother or the baby.

Both the initial and simplified models demonstrated excellent discrimination (optimism-adjusted C-statistic 0.873 in the initial model and 0.885 in the simplified model) and good calibration. The calibration plot indicated that model predictions closely matched observed outcomes across risk groups. To provide a more clinically intuitive summary, we examined how well the simplified model’s predictions aligned with actual outcomes at a 50% probability threshold. Presenting the results in this way provides a clearer clinical perspective: if the model predicts survival, almost 93% of infants do in fact survive, whereas if it predicts death, about two-thirds of infants die. This highlights that the model is most reliable when predicting survival, while predictions of death are less certain but still useful to inform counselling conversations with families. These findings suggest that the model may be most helpful in reassuring families when survival is predicted, while still offering valuable, though less certain, guidance when death is predicted.

### Comparison to previous research

This model showed improved discrimination compared to previous models [6, 7]. Ambalavanan et al. developed a prediction model for neonatal death in infants with neonatal encephalopathy, achieving an AUC of 0.81 [6]. Similar to the present study, their model identified base deficit >22 mmol/L as a significant predictor of neonatal mortality. While the authors also used variables routinely collected during the early neonatal resuscitation period, their chosen predictors differed from those in our study (decerebrate posture, absent suck, absence of antepartum haemorrhage and base deficit of first postnatal gas > 22 mmol/L). Significantly, this model was based on American data collected over 20 years ago and requires updating to reflect current clinical practices and populations. Additionally, the study did not include a sample size calculation, nor did it perform internal validation or apply methods to adjust for overfitting.

Tegegne et al. developed a prediction model to predict neonatal mortality in asphyxiated neonates admitted to the neonatal intensive care unit [7]. The model achieved an optimism-adjusted C-statistic of 0.775. This study was conducted in a low-income country, where perinatal care practices and outcomes may differ significantly from those in high-income settings. Notably, this study had a high incidence of neonatal mortality (27.2% versus 11.9% in the present study).

Lew et al. developed a deep learning algorithm that combined MRI and clinical data to predict 2-year neurodevelopmental outcomes in neonates with hypoxic-ischaemic encephalopathy [8]. Their model demonstrated strong discriminative performance for predicting mortality alone, achieving an AUC of 0.92. While MRI data are valuable, it is important to note that these data are not available until several days of age [26, 27]. In contrast, our prediction model is based on readily available clinical and biochemical data available within the first day of life, providing a practical tool to improve parental counselling.

### Clinical implications

Clear and compassionate communication with families is essential in the early days following birth, when uncertainty about outcomes can significantly contribute to parental distress [5]. This study provides an evidence-based tool to predict neonatal mortality in infants with neonatal encephalopathy undergoing TH using routinely available neonatal data. The model is intended solely for parental counselling and was not designed to determine whether TH should be initiated or continued. If a model were to be developed for clinical decision-making, it would need to undergo not only external validation but also prospective evaluation and formal impact assessment to determine whether its use improves clinical decision-making and patient outcomes in routine clinical practice [28, 29].

The model’s excellent discriminative ability (optimism-adjusted C-statistic 0.885) supports its utility in guiding parental counselling. Furthermore, the inclusion of readily available clinical and biochemical variables ensures the model’s practical application in similar real-world high-income settings. The THERM tool, implemented as a simple and interactive Excel-based calculator, allows clinicians to quickly generate individualised risk estimates before family meetings.

### Strengths and limitations

Our study contained several strengths. First, we used a comprehensive national dataset, incorporating data from 19 maternity units over a 6-year period. This ensures that the findings are nationally inclusive, enhancing the generalisability of the results to similar healthcare settings. Second, our predictors are routinely collected. This practical approach ensures that the model can be readily implemented in clinical settings without requiring additional, specialised resources or equipment, thereby enhancing its utility in everyday clinical practice.

Third, we created a user-friendly interactive tool to calculate individual neonatal mortality risk. Dropdown menus for categorical variables and clear variable definitions enhance usability and reduce the likelihood of data entry errors [30]. This tool provides a practical and user-friendly approach for applying the prediction model in clinical settings, enabling clinicians to make individualized, evidence-based assessments of neonatal mortality risk.

Fourth, this study adhered to the TRIPOD guidelines, ensuring transparency and reproducibility of the research.

Some limitations should be noted. First, as a retrospective study, this analysis is inherently subject to limitations, particularly related to missing data. However, the proportion of missing data in this study was relatively low at 5.17% for the simplified model and 6.90% for the initial model, which is below the 10% threshold associated with a high risk of bias [31]. Furthermore, since the data satisfy the MCAR assumption, the missing data can be considered a random subset of the complete dataset [32]. Consequently, excluding cases with missing data under the MCAR assumption is unlikely to introduce bias into the findings [32]. Second, “seizures during the first day of life” was included in the model. While neonatal seizures can be challenging to recognise clinically, the variable is included in many candidacy checklists for TH [15, 33]. The model assumes experienced clinicians will assess it, as its purpose is to support parental counselling during initial neonatal resuscitation. We acknowledge that continuous EEG monitoring, the gold standard for seizure detection, was not uniformly available across all centres, and subclinical seizures may therefore have been missed. This limitation could contribute to misclassification, although we attempted to reduce this risk by requiring documentation of clinical seizure activity, EEG changes where available, and antiepileptic treatment. Third, the model was developed using a recent, nationally representative dataset from a high-income setting, which enhances generalisability to similar healthcare environments. Nonetheless, temporal and geographical external validation will be required to assess performance and applicability in other settings with different healthcare resources and practices. However, it is recommended that an independent research team conduct external validation to evaluate performance objectively [34]. Therefore, we have included the values to calculate the linear predictor of our model, allowing researchers to conduct independent external validation (Supplementary Material 3).

Fourth, although the data extraction form used for national data collection was not formally validated, it was employed by expert clinicians in obstetrics and neonatology and was overseen by the National TH Coordinator and the Therapeutic Hypothermia Steering Committee to ensure the accuracy of the information.

Fifth, although TH is of uncertain benefit in infants born at 34–35 weeks, we retained these cases to reflect real-world clinical practice, where TH may be used in late preterm infants [35, 36]. Their small number (*n* = 11, 2.7%) makes it unlikely that their inclusion influenced the model’s performance meaningfully.

Sixth, this study used unadjusted logistic regression as part of the predictor selection process, in addition to expert input from neonatologists and obstetricians, and a review of the existing literature, while also considering data availability. While this is a widely used method, there is an argument that it may lead to the exclusion of predictors that, while not individually significant, could provide valuable information when considered in combination with other variables [37]. However, this method has been used in previously published similar research, and there is no universally accepted standard for predictor selection [38–41]. This approach allowed us to explore associations between individual predictors and neonatal mortality in a national cohort.

Seventh, although methods such as machine learning are increasingly used in prediction modelling, we selected logistic regression because it is transparent, easy to interpret, and widely applied in similar research [36, 42–44]. It is also recognised in methodological guidance as a common starting point for binary outcomes [37]. Importantly, this approach allowed us to translate the model into a simple tool for use in clinical practice.

Eighth, long-term neurodevelopmental outcomes, which are highly relevant for families, were beyond the scope of this study but should be addressed in future research.

Lastly, although the final model met the conventional events per parameter threshold of 10 (11.5 events per parameter), the modest number of deaths (*n* = 46) may still limit stability. Internal validation using bootstrapping was therefore undertaken. This approach quantifies and corrects for optimism, yielding adjusted estimates of discrimination and calibration that better reflect expected performance in new populations. Nonetheless, external validation remains essential.

## Conclusion

This study developed and internally validated a risk prediction model for neonatal mortality in infants with neonatal encephalopathy undergoing therapeutic hypothermia, using national data from 19 maternity hospitals and units across the Republic of Ireland. By incorporating four routinely collected clinical and biochemical variables, the model demonstrated excellent discriminative ability (optimism-adjusted C-statistic, 0.885) and good calibration. The use of our prediction tool (THERM) could potentially assist with individualised risk assessment for neonatal mortality and aid in parental counselling in this setting. External validation and prospective studies are needed to confirm its utility in clinical practice.

## Supplementary information

Appendix: Development of a Prediction Model for Mortality in Infants Undergoing Therapeutic Hypothermia for Neonatal Encephalopathy

Data Set 1

Data Set 2

## Data Availability

The datasets analysed during the current study are available from the corresponding author on reasonable request.
