# Supplementary material for: Development of a prediction model for mortality in infants undergoing therapeutic hypothermia for neonatal encephalopathy
Source: J Perinatol. 2026 Jan 5;46(6):934–41. doi: 10.1038/s41372-025-02547-z (PMC13290479; doi:10.1038/s41372-025-02547-z)
Supplement: Supplementary file 1 — Appendix: Development of a Prediction Model for Mortality in Infants Undergoing Therapeutic Hypothermia for Neonatal Encephalopathy [file 41372_2025_2547_MOESM1_ESM.docx]

**Appendix**

**Article:** Development of a Prediction Model for Mortality in Infants Undergoing Therapeutic Hypothermia for Neonatal Encephalopathy

[Appendix A1. Model performance metrics 1](#_Toc209699886)

[Table A1. Maternal demographics and medical history 2](#_Toc209699887)

[Table A2. Labour and delivery details 4](#_Toc209699888)

[Table A3. Neonatal resuscitation and biochemical characteristics 8](#_Toc209699889)

[Table A4. Crosstabulation of mode of delivery and acute perinatal events 10](#_Toc209699890)

[Table A5. Unadjusted analysis of the association between maternal characteristics, labour and delivery outcomes, and resuscitation and biochemical characteristics and neonatal mortality in infants undergoing therapeutic hypothermia 10](#_Toc209699891)

[Table A6. Multivariable analysis including the variable meconium 15](#_Toc209699892)

[Figure A1. Calibration plot of observed against expected probabilities across ten risk groups of individuals (initial prediction model) 16](#_Toc209699893)

[Figure A2. Calibration plot of observed against expected probabilities across ten risk groups of individuals (simplified prediction model) 17](#_Toc209699894)

[Table A7. Confusion matrix of predicted versus observed outcomes using the simplified prediction model (50% probability threshold) 17](#_Toc209699895)

# Appendix A1. Model performance metrics

The Brier score ranges from 0 to 1, with lower values indicating better performance^23^. Although its expected value lies between 0 and 1, the score is not standardised, so there is no universally accepted threshold for a good Brier score. In practice, a useful risk prediction model should have a Brier score no higher than 0.25. This threshold corresponds to a non-informative model with a 50% incidence of the outcome^24, 25^. Cragg & Uhler’s R² is a generalised version of the R² used in linear regression, helping to quantify the predictive strength of a model^26^. A higher value (closer to 1) indicates that the model performs well compared to the null model, while a value near 0 suggests the model provides little improvement over basic prediction^27^. In logistic regression, Cragg & Uhler’s R² values are typically lower than in linear models. Values between 0.3 and 0.7 suggest reasonable explanatory power, while values closer to 1 are rare and may indicate overfitting if not externally validated^28^. Discrimination refers to the model’s ability to differentiate between patients who experience the outcome and those who do not.^29^ Calibration reflects how closely the model’s predictions match observed outcomes.^29^

# Table A1. Maternal demographics and medical history

| Variable | All N | Neonatal Death | | p-value |
| --- | --- | --- | --- | --- |
|  | **N (%)** | **No**  **N(%)** | **Yes**  **N(%)** |  |
| Parity | 385 | 339 | 46 | 0.319 |
| Primiparous | 228 (58.7) | 197 (58.1) | 29 (63.0) |  |
| Multiparous | 159 (41.3) | 142 (41.9) | 17 (37.0) |  |
| Not documented | 0 |  |  |  |
| Body Mass Index | 369 | 323 | 46 | 0.352 |
| ≤24.9 kg/m^2^ | 143 (38.8) | 129 (39.9) | 14 (30.4) |  |
| 25.0 – 29.9 kg/m^2^ | 119 (32.2) | 104 (32.2) | 15 (32.6) |  |
| ≥30 kg/m^2^ | 107 (29.0) | 90 (27.9) | 17 (37.0) |  |
| Not documented | 16 (4.2) |  |  |  |
| Ethnicity | 382 | 336 | 46 | 0.156 |
| White Irish | 300 (78.5) | 266 (79.2) | 34 (73.9) |  |
| White Non-Irish | 42 (11.0) | 37 (11.0) | 5 (10.9) |  |
| Asian | 17 (4.5) | 12 (3.6) | 5 (10.9) |  |
| Black or other | 23 (6.0) | 21 (6.3) | 2 (4.3) |  |
| Not documented | 3 (0.8) |  |  |  |
| Employment status | 359 | 316 | 43 | 0.695 |
| Employed | 273 (76.0) | 241 (76.3) | 32 (74.4) |  |
| Unemployed or student | 56 (15.6) | 50 (15.8) | 6 (14.0) |  |
| Homemaker | 30 (8.4) | 25 (7.9) | 5 (11.6) |  |
| Not documented | 26 (6.8) |  |  |  |
| Smoking status at booking | 380 | 334 | 46 | 0.190 |
| Smoker | 47 (12.4) | 39 (11.7) | 8 (17.4) |  |
| Non-smoker | 333 (87.6) | 295 (88.3) | 38 (82.6) |  |
| Not documented | 5 (1.3) |  |  |  |
| Maternal medical condition in current pregnancy* | 385 | 339 | 46 | 0.497 |
| Yes | 80 (20.8) | 70 (20.6) | 10 (21.7) |  |
| No | 305 (79.2) | 269 (79.4) | 36 (78.3) |  |
| Not Documented | 0 |  |  |  |
| Previous Caesarean section | 383 | 337 | 46 | 0.411 |
| Yes | 27 (7.0) | 23 (6.8) | 4 (8.7) |  |
| No | 356 (93.0) | 314 (93.2) | 42 (91.3) |  |
| Not documented | 2 (0.5) |  |  |  |
| Maternal Age | Median | Interquartile Range |  |  |
| N=384 | 33 years | 7 |  |  |

*Maternal medical conditions in the current pregnancy, defined as hypertensive disorders (including pre-eclampsia, pregnancy induced hypertension and essential hypertension), diabetes mellitus (either gestational or pre-existing) or thyroid disease (hypothyroidism or hyperthyroidism).

# Table A2. Labour and delivery details

| Variable | All | Neonatal Death | | p-value |
| --- | --- | --- | --- | --- |
|  | N (%) | **No**  **N(%)** | **Yes**  **N(%)** |  |
| Onset of labour | 385 | 339 | 46 | 0.102 |
| Spontaneous | 167 (43.4) | 151 (44.5) | 16 (34.8) |  |
| Induction | 138 (35.8) | 123 (36.3) | 15 (32.6) |  |
| Never in labour | 80 (20.8) | 65 (19.2) | 15 (32.6) |  |
| Not documented | 0 |  |  |  |
| Use of oxytocin | 375 | 331 | 44 | **0.033** |
| Yes | 126 (33.6) | 117 (35.3) | 9 (20.5) |  |
| No | 249 (66.4) | 214 (64.7) | 35 (79.5) |  |
| Not documented | 10 (2.6) |  |  |  |
| Pyrexia in labour | 385 | 339 | 46 | 0.371 |
| Yes | 44 (11.4) | 40 (11.8) | 4 (8.7) |  |
| No | 341 (88.6) | 299 (88.2) | 42 (91.3) |  |
| Not documented | 0 |  |  |  |
| Meconium | 322 | 290 | 32 | 0.182 |
| Yes | 103 (32.0) | 90 (31.0) | 13 (40.6) |  |
| No | 219 (68.0) | 200 (69.0) | 19 (59.4) |  |
| Not documented | 63 (16.4) |  |  |  |
| Gestational age at delivery | 384 | 338 | 46 | 0.417 |
| <37 weeks | 33 (8.6) | 27 (8.0) | 6 (13.0) |  |
| 37 – 40+6 weeks | 261 (68.0) | 233 (68.9) | 28 (60.9) |  |
| ≥41 weeks | 90 (23.4) | 78 (23.1) | 12 (26.1) |  |
| Not documented | 1 (0.3) |  |  |  |
| Sex of infant | 385 | 339 | 46 | **0.013** |
| Male | 214 (55.6) | 196 (57.8) | 18 (39.1) |  |
| Female | 171 (44.4) | 143 (42.2) | 28 (60.9) |  |
| Not documented | 0 |  |  |  |
| Birth Weight | 385 | 339 | 46 | 0.249 |
| ≤2499g | 27 (7.0) | 25 (7.4) | 2 (4.3) |  |
| 2500 – 2999g | 69 (17.9) | 59 (17.4) | 10 (21.7) |  |
| 3000 – 3499g | 106 (27.5) | 88 (26.0) | 18 (39.1) |  |
| 3500 – 3999g | 118 (30.6) | 107 (31.6) | 11 (23.9) |  |
| ≥4000g | 65 (16.9) | 60 (17.7) | 5 (10.9) |  |
| Not documented | 0 |  |  |  |
| Pregnancy Type | 385 | 339 | 46 | 0.227 |
| Multiple pregnancy | 14 (3.6) | 11 (3.2) | 3 (6.5) |  |
| Singleton pregnancy | 371 (96.4) | 328 (96.8) | 43 (93.5) |  |
| Not documented | 0 |  |  |  |
| Mode of delivery | 385 | 339 | 46 | **0.005** |
| Spontaneous vaginal | 92 (23.9) | 84 (24.8) | 8 (17.4) |  |
| Operative vaginal | 123 (31.9) | 116 (34.2) | 7 (15.2) |  |
| Prelabour Caesarean section | 82 (21.3) | 65 (19.2) | 17 (37.0) |  |
| Caesarean section after onset of labour | 88 (22.9) | 74 (21.8) | 14 (30.4) |  |
| Not documented | 0 |  |  |  |
| Birth Status | 385 | 339 | 46 | 0.438 |
| Inborn | 251 (65.2) | 220 (64.9) | 31 (67.4) |  |
| Outborn | 134 (34.8) | 119 (35.1) | 15 (32.6) |  |
| Not documented | 0 |  |  |  |
| Occurrence of an acute perinatal event* | 385 | 339 | 46 | 0.070 |
| Yes | 289 (75.1) | 250 (73.7) | 39 (84.8) |  |
| No | 96 (24.9) | 89 (26.3) | 7 (15.2) |  |
| Not documented | 0 |  |  |  |
| Clinical suspicion of placental abruption | 339 | 294 | 45 | 0.112 |
| Yes | 32 (9.4) | 25 (8.5) | 7 (15.6) |  |
| No | 307 (90.6) | 269 (91.5) | 38 (84.4) |  |
| Not documented | 46 (11.9) |  |  |  |
| Time of Day (Birth) | 385 | 339 | 46 | 0.106 |
| 0800h to 1959h | 172 (44.7) | 147 (43.4) | 25 (54.3) |  |
| 2000h to 0759h | 213 (55.3) | 192 (56.6) | 21 (45.7) |  |
| Not documented | 0 |  |  |  |
| Day of Week (Birth) | 385 | 339 | 46 | 0.169 |
| Monday to Friday | 292 (75.8) | 254 (74.9) | 38 (82.6) |  |
| Saturday or Sunday | 93 (24.2) | 85 (25.1) | 8 (17.4) |  |
| Not documented | 0 |  |  |  |

*Acute perinatal event defined as: umbilical cord prolapse, uterine rupture, antenatal haemorrhage, shoulder dystocia or severe fetal heart rate abnormality^4^.

# Table A3. Neonatal resuscitation and biochemical characteristics

| Variable | All | Neonatal Death | | p-value |
| --- | --- | --- | --- | --- |
|  | N (%) | No  N (%) | Yes  N (%) |  |
| 5-minute Apgar score | 381 | 335 | 46 | **<0.001** |
| <3 | 100 (26.2) | 69 (20.6) | 31 (67.4) |  |
| ≥3 | 281 (73.8) | 266 (79.4) | 15 (32.6) |  |
| Not documented | 4 (1.0) |  |  |  |
| Establishment of Spontaneous Respiration | 385 | 339 | 46 | **<0.001** |
| Yes | 192 (49.9) | 185 (54.6) | 7 (15.2) |  |
| No | 193 (50.1) | 154 (45.4) | 39 (84.8) |  |
| Not documented | 0 |  |  |  |
| Assisted ventilation for ≥ 10 minutes from birth | 382 | 336 | 46 | **<0.001** |
| Yes | 244 (63.9) | 201 (59.8) | 43 (93.5) |  |
| No | 138 (36.1) | 135 (40.2) | 3 (6.5) |  |
| Not documented | 3 (0.8) |  |  |  |
| Use of Adrenaline | 385 | 339 | 46 | **<0.001** |
| Yes | 65 (16.9) | 34 (10.0) | 31 (67.4) |  |
| No | 320 (83.1) | 305 (90.0) | 15 (32.6) |  |
| Not documented | 0 |  |  |  |
| Chest compressions | 384 | 338 | 46 | **<0.001** |
| Yes | 129 (33.6) | 93 (27.5) | 36 (78.3) |  |
| No | 255 (66.4) | 245 (72.5) | 10 (21.7) |  |
| Not documented | 1 (0.3) |  |  | **<0.001** |
| Intubation | 385 | 339 | 46 |  |
| Yes | 234 (60.8) | 194 (57.2) | 40 (87.0) |  |
| No | 151 (39.2) | 145 (42,8) | 6 (13.0) |  |
| Not documented | 0 |  |  |  |
| Seizure during first day of life | 385 |  |  | **<0.001** |
| Yes | 122 (31.7) | 89 (26.3) | 33 (71.7) |  |
| No | 263 (68.3) | 250 (73.7) | 13 (28.3) |  |
| Not documented | 0 |  |  |  |
| pH* | 383 | 339 | 44 | **<0.001** |
| ≤6.70 | 44 (11.5) | 30 (8.8) | 14 (31.8) |  |
| >6.70 | 339 (88.5) | 309 (91.2) | 30 (68.2) |  |
| Not documented | 2 (0.5) |  |  |  |
| Base excess* | 385 | 339 | 46 | **<0.001** |
| ≤ -22 mmol/L | 63 (16.4) | 36 (10.6) | 27 (58.7) |  |
| > -22 mmol/L | 322 (83.6) | 303 (89.4) | 19 (41.3) |  |
| Not documented | 0 |  |  |  |

*Cord blood or first infant blood gas within the first hour of life

# Table A4. Crosstabulation of mode of delivery and acute perinatal events

| Variable | | All  N (%) | Mode of Delivery | | | | p-value |
| --- | --- | --- | --- | --- | --- | --- | --- |
|  | |  | **SVD** | **OVD** | **Prelabour CS** | **CS after onset of labour** |  |
| Acute Perinatal Event* | | 385 | 92 | 123 | 82 | 88 | **<0.001** |
| Yes | N(%) | 289 (75.1) | 42 (45.7) | 98 (79.7) | 74 (90.2) | 75 (85.2) |  |
| No | N(%) | 96 (24.9) | 50 (54.3) | 25 (20.3) | 8 (9.8) | 13 (14.8) |  |

Key: SVD = spontaneous vaginal delivery, OVD = operative vaginal delivery, CS = Caesarean section

*Acute perinatal event defined as: umbilical cord prolapse, uterine rupture, antenatal haemorrhage, shoulder dystocia or severe fetal heart rate abnormality^4^.

# Table A5. Unadjusted analysis of the association between maternal characteristics, labour and delivery outcomes, and resuscitation and biochemical characteristics and neonatal mortality in infants undergoing therapeutic hypothermia

| Characteristic | N (%) | OR (95% CI) | P-value |  |
| --- | --- | --- | --- | --- |
| Parity | 406 |  |  |  |
| Nulliparous | 236 (58.1) | Ref |  |  |
| Multiparous | 170 (41.9) | 0.88 (0.48, 1.61) | 0.68 |  |
| Body Mass Index | 390 |  |  |  |
| ≤24.9 kg/m^2^ | 153 (39.2) | Ref | 0.38 |  |
| 25.0 – 29.9 kg/m^2^ | 127 (32.6) | 1.32 (0.64, 2.74) | 0.45 |  |
| ≥30 kg/m^2^ | 110 (28.2) | 1.68 (0.81, 3.45) | 0.16 |  |
| Employment status | 379 |  |  |  |
| Employed | 284 (74.9) | Ref | 0.90 |  |
| Unemployed or student | 62 (16.4) | 1.05 (0.46, 2.40) | 0.90 |  |
| Homemaker | 33 (8.7) | 1.27 (0.46, 3.51) | 0.64 |  |
| Smoking status at booking | 401 |  |  |  |
| Non-smoker | 350 (87.3) | Ref |  |  |
| Smoker | 51 (12.7) | 1.33 (0.59, 3.01) | 0.50 |  |
| Maternal condition in current pregnancy* | 406 |  |  |  |
| No | 322 (79.3) | Ref |  |  |
| Yes | 84 (20.7) | 0.93 (0.44, 1.94) | 0.84 |  |
| Previous Cesarean Section | 403 |  |  |  |
| No | 375 (93.1) | Ref |  |  |
| Yes | 28 (6.9) | 1.16 (0.39, 3.50) | 0.79 |  |
| Meconium | 339 |  |  |  |
| No | 231 (68.1) | Ref |  |  |
| Yes | 108 (31.9) | 1.84 (0.91, 3.70) | 0.09 |  |
| Gestational age at delivery | 404 |  |  |  |
| <37 weeks | 34 (8.4) | Ref | 0.41 |  |
| 37 – 40+6 weeks | 277 (68.6) | 0.59 (0.23, 1.53) | 0.28 |  |
| ≥41 weeks | 93 (23.0) | 0.83 (0.29, 2.36) | 0.72 |  |
| Sex of infant | 406 |  |  |  |
| Male | 255 (62.8) | Ref |  |  |
| Female | 181 (44.6) | 1.76 (0.97, 3.18) | 0.06 |  |
| Birth Weight | 406 |  |  |  |
| ≤2499g | 29 (71.4) | Ref | 0.24 |  |
| 2500 – 2999g | 74 (18.2) | 2.61 (0.55, 12.48) | 0.23 |  |
| 3000 – 3499g | 112 (30.0) | 2.76 (0.60, 12.59) | 0.19 |  |
| 3500 – 3999g | 126 (31.0) | 1.55 (0.33, 7.29) | 0.58 |  |
| ≥4000g | 65 (16.0) | 1.13 (0.21, 6.17) | 0.89 |  |
| Mode of delivery | 406 |  |  |  |
| Prelabour Caesarean section | 86 (21.2) | Ref | **0.01** |  |
| Caesarean section after onset of labour | 89 (21.9) | 0.66 (0.31, 1.41) | 0.28 |  |
| Spontaneous vaginal delivery | 102 (25.1) | 0.38 (1.68, 0.88) | **0.02** |  |
| Operative vaginal delivery | 128 (31.5) | 0.24 (0.10, 0.57) | **0.001** |  |
| Birth Status | 406 |  |  |  |
| Inborn | 262 (64.5) | Ref |  |  |
| Outborn | 144 (35.5) | 1.2 (0.66, 2.20) | 0.55 |  |
| Occurrence of an acute perinatal event** | 406 |  |  |  |
| No | 103 (25.4) | Ref |  |  |
| Yes | 303 (74.6) | 1.96 (0.89, 4.33) | 0.09 |  |
| Time of Day (Birth) | 406 |  |  |  |
| 0800h to 2000h | 180 (44.3) | Ref |  |  |
| 2001h to 0759h | 226 (55.7) | 0.62 (0.34, 1.11) | 0.11 |  |
| Day of Week (Birth) | 406 |  |  |  |
| Monday to Friday | 309 (76.1) | Ref |  |  |
| Saturday or Sunday | 97 (23.9) | 0.56 (0.25, 1.23) | 0.15 |  |
| 5-minute Apgar score | 399 |  |  |  |
| ≥3 | 296 (74.2) | Ref |  |  |
| <3 | 103 (25.8) | 6.96 (3.69, 13.12) | **<0.001** |  |
| Establishment of spontaneous respiration | 403 |  |  |  |
| Yes | 204 (50.6) | Ref |  |  |
| No | 199 (49.4) | 5.03 (2.44, 10.37) | **<0.001** |  |
| Use of Adrenaline | 404 |  |  |  |
| No | 338 (83.7) | Ref |  |  |
| Yes | 66 (16.3) | 16.73 (8.50, 32.93) | **<0.001** |  |
| Chest compressions | 401 |  |  |  |
| No | 131 (32.7) | Ref |  |  |
| Yes | 270 (67.3) | 8.79 (4.40, 17.53) | **<0.001** |  |
| Intubation | 402 |  |  |  |
| No | 241 (60.0) | Ref |  |  |
| Yes | 161 (40.0) | 4.04 (1.84, 8.85) | **<0.001** |  |
| pH ≤6.70*** | 394 |  |  |  |
| No | 350 (88.8) | Ref |  |  |
| Yes | 44 (11.2) | 4.48 (2.16, 9.29) | **<0.001** |  |
| Base excess ≤ -22 mmol/L*** | 386 |  |  |  |
| No | 323 (83.7) | Ref |  |  |
| Yes | 63 (16.3) | 11.36 (5.79, 22.29) | **<0.001** |  |
| Seizure during day one of life | 405 |  |  |  |
| No | 277 (68.4) | Ref |  |  |
| Yes | 128 (31.6) | 5.90 (3.11, 11.18) | **<0.001** |  |
| Maternal Age (years) | 406 | 1.03 (0.97, 1.09) | 0.30 |  |
| ≤24 | 48 (11.8) | Ref | 0.26 |  |
| 25-34 | 216 (53.2) | 1.28 (0.47, 3.51) | 0.63 |  |
| 35-39 | 112 (27.6) | 0.94 (0.31, 2.86) | 0.91 |  |
| ≥40 | 30 (7.4) | 2.62 (0.75, 9.18) | 0.13 |  |

*Maternal medical conditions in the current pregnancy, defined as hypertensive disorders (including pre-eclampsia, pregnancy induced hypertension and essential hypertension), diabetes mellitus (either gestational or pre-existing) or thyroid disease (hypothyroidism or hyperthyroidism).

**Acute perinatal event defined as: umbilical cord prolapse, uterine rupture, antenatal haemorrhage, shoulder dystocia or severe fetal heart rate abnormality^4^.

***Cord blood or first infant blood gas within the first hour of life

# Table A6. Multivariable analysis including the variable meconium

| Variable | Coefficient (95% CI) | N (%) | OR (95% CI) |
| --- | --- | --- | --- |
| Use of adrenaline |  |  |  |
| No | - | 338 (83.3) | Ref |
| Yes | 1.81 (0.85, 2.77) | 66 (16.3) | 6.90 (2.66, 17.90) |
| Base excess ≤ -22 mmol/L |  |  |  |
| No | - | 323 (79.6) | Ref |
| Yes | 1.81 (0.85, 2.77) | 66 (16.3) | 6.11 (2.34, 15.96) |
| Seizures on First Day of Life |  |  |  |
| No | - | 277 (68.2) | Ref |
| Yes | 1.63 (0.60, 2.67) | 128 (31.5) | 5.13 (1.82, 14.42) |
| Constant | -4.23 (-5.17, -3.29) |  | 0.01 (0.01, 0.04) |

# Figure A1. Calibration plot of observed against expected probabilities across ten risk groups of individuals (initial prediction model)

# Figure A2. Calibration plot of observed against expected probabilities across ten risk groups of individuals (simplified prediction model)

# Table A7. Confusion matrix of predicted versus observed outcomes using the simplified prediction model (50% probability threshold)

|  | Predicted survive (≤50%) | Predicted die (>50%) | Total |
| --- | --- | --- | --- |
| Actually survived | 329 | 10 | 339 |
| Actually died | 25 | 21 | 46 |
| Total | 354 | 31 | 385 |

Table A7 is based on the simplified prediction model, which was selected for clinical applicability.
